# Positive Childhood Experiences and Adult Mental and Relational Health in a Statewide Sample: Associations Across Adverse Childhood Experiences Levels

**DOI:** 10.1001/jamapediatrics.2019.3007

**Published:** 2019-09-09

**Authors:** Christina Bethell, Jennifer Jones, Narangerel Gombojav, Jeff Linkenbach, Robert Sege

**Affiliations:** 1 Johns Hopkins Bloomberg School of Public Health and Child and Adolescent Health Measurement Initiative, Baltimore, Maryland; 2 Alliance for Strong Families and Communities, Milwaukee, Wisconsin; 3 The Montana Institute, Bozeman, Montana; 4 Institute for Clinical Research and Health Policy Studies, Tufts Medical Center, Boston, Massachusetts

## Abstract

**Importance:**

Associations between adverse childhood experiences (ACEs) and risks for adult depression, poor mental health, and insufficient social and emotional support have been documented. Less is known about how positive childhood experiences (PCEs) co-occur with and may modulate the effect of ACEs on adult mental and relational health.

**Objective:**

To evaluate associations between adult-reported PCEs and (1) adult depression and/or poor mental health (D/PMH) and (2) adult-reported social and emotional support (ARSES) across ACEs exposure levels.

**Design, Setting, and Participants:**

Data were from the cross-sectional 2015 Wisconsin Behavioral Risk Factor Survey, a random digit-dial telephone survey of noninstitutionalized Wisconsin adults 18 years and older (n = 6188). Data were weighted to be representative of the entire population of Wisconsin adults in 2015. Data were analyzed between September 2016 and January 2019.

**Main Outcomes and Measures:**

The definition of D/PMH includes adults with a depression diagnosis (ever) and/or 14 or more poor mental health days in the past month. The definition of PCEs includes 7 positive interpersonal experiences with family, friends, and in school/the community. Standard Behavioral Risk Factor Survey ACEs and ARSES variables were used.

**Results:**

In the 2015 Wisconsin Behavioral Risk Factor Survey sample of adults (50.7% women; 84.9% white), the adjusted odds of D/PMH were 72% lower (OR, 0.28; 95% CI, 0.21-0.39) for adults reporting 6 to 7 vs 0 to 2 PCEs (12.6% vs 48.2%). Odds were 50% lower (OR, 0.50; 95% CI, 0.36-0.69) for those reporting 3 to 5 vs 0 to 2 PCEs (25.1% vs 48.2%). Associations were similar in magnitude for adults reporting 1, 2 to 3, or 4 to 8 ACEs. The adjusted odds that adults reported “always” on the ARSES variable were 3.53 times (95% CI, 2.60-4.80) greater for adults with 6 to 7 vs 0 to 2 PCEs. Associations for 3 to 5 PCEs were not significant. The PCE associations with D/PMH remained stable across each ACEs exposure level when controlling for ARSES.

**Conclusions and Relevance:**

Positive childhood experiences show dose-response associations with D/PMH and ARSES after accounting for exposure to ACEs. The proactive promotion of PCEs for children may reduce risk for adult D/PMH and promote adult relational health. Joint assessment of PCEs and ACEs may better target needs and interventions and enable a focus on building strengths to promote well-being. Findings support prioritizing possibilities to foster safe, stable nurturing relationships for children that consider the health outcomes of positive experiences.

## Key Points

**Question:**

Are positive childhood experiences (PCEs) associated with adult depression and/or poor mental health (D/PMH) and adult-reported social and emotional support (ARSES) independent from adverse childhood experiences (ACEs)?

**Findings:**

In this cross-sectional study, adults reporting higher PCEs had lower odds of D/PMH and greater ARSES after accounting for ACEs. The associations of PCEs with D/PMH also remained stable when controlling for ARSES.

**Meaning:**

Positive childhood experiences demonstrate a dose-response association with adult D/PMH and ARSES after adjustment for ACEs; assessing and proactively promoting PCEs may reduce adult mental and relational health problems, even in the concurrent presence of ACEs.

## Teaser

This study evaluates associations between adult-reported positive childhood experiences and adult depression and/or poor mental health as well as reported social and emotional support across adverse childhood experience exposures levels.

## Introduction

Research demonstrates that both positive and adverse experiences shape brain development and health across the life span.^1,2,3,4,5^^.^ Understanding human development requires a model that incorporates both risks (factors that decrease the likelihood of successful development) and opportunities (factors that increase the likelihood of successful development). On the positive side, successful child development depends on secure attachment during the first years of life.^6,7^ As the child grows, exposure to spoken language^8^ and having the presence of safe, stable, nurturing relationships and environments are important factors for optimal development.^9,10^ On the other hand, children with adverse childhood experiences (ACEs) are at risk for observable changes in brain anatomy,^11^ gene expression,^12,13^ and delays in social, emotional, physical, and cognitive development lasting into adulthood.^3,4,5,14,15,16,17^

According to standardized measures, an estimated 61.5% of adults^18^ and 48% of children^19^ in the United States have been exposed to ACEs, with more than one-third of these having multiple exposures.^18,19^ The wide-ranging negative associations between exposure to multiple ACEs and diminished adult and child health are well documented.^14,19,20,21,22^ Most notable is the especially strong evidence linking ACEs with adult mental health problems including depression.^22,23,24,25,26,27,28^ A robust literature also exists regarding the effect of ACEs on adult relational health (often assessed by whether adults report that they get the social and emotional support they need) and how diminished adult social and emotional support contributes to poorer adult physical and mental health.^29,30,31,32,33,34,35,36,37,38,39,40,41,42,43,44,45,46,47,48,49,50,51,52,53,54,55,56^

Beyond the extensive and growing body of research dealing with lifelong correlates of adversity, many prior studies identify resiliency factors and adaptive skills and interventions associated with improved child development and child and adult health outcomes.^2,3,16,17,25,26,27,28,29,30,31,32,33,34,35,36,37,38,39,40,41,42,43,44,45,46,47,48,49,50,51,52,53,54,55^ For example, the Search Institute developed a list of “40 Developmental Assets” and demonstrated associations between the number of assets and both positive and negative outcomes.^52^ A national population-based study^53^ on child flourishing and resilience shows strong associations with levels of family resilience and parent-child connection for children with exposures to greater ACEs, poverty, and chronic conditions. Similar studies, such as those assessing the US Centers for Disease Control and Prevention (CDC)’s “safe, stable, and nurturing relationships” model, show similar findings.^55^

Despite these advances, standardized measures and the prevalence of positive childhood experiences (PCEs) at the population level for adults or children are still unknown. Yet prior studies, using data from small or nonrepresentative samples, have explored interactions between PCEs and ACEs.^25,41,56^ For example, 1 study,^41^ conducted by Kaiser Permanente and CDC investigators, analyzed a cohort of 4648 women. They found that adult reports of specific positive family experiences in childhood (including closeness, support, loyalty, protection, love, importance, and responsiveness to health needs) were associated with lower rates of adolescent pregnancy across all ACEs exposure levels.^41^ The protective effects of reported interpersonal PCEs against mental health problems in adulthood have also been found among pregnant women^25^ and young adults^56^ exposed to ACEs. Despite these findings, few subsequent studies on ACEs have simultaneously evaluated PCEs.

Collectively, prior studies on child development point to the importance of research focusing on PCEs, especially those associated with parent-child attachment, positive parenting (eg, parental warmth, responsiveness, and support), family health, and positive relationships with friends, in school, and in the community. Knowledge of whether retrospectively reported PCEs co-occur with ACEs and how PCEs interact with ACEs to effect adult mental and relational health is needed to inform the nation’s growing focus on addressing early life and social determinants of healthy development and lifelong health.

This study used data from the 2015 Wisconsin Behavioral Risk Factor Survey (WI BRFS), a representative, population-based survey,^57^ to assess the prevalence of PCEs in an adult sample and evaluate hypothesized associations with adult mental and relational health across 4 ACEs exposure levels. This study builds on a 2017 *Health Outcomes of Positive Experiences* report^58^ featuring bivariate findings from the 2015 WI BRFS associating individual PCEs with negative adult health outcomes. Here, we construct a PCEs cumulative score measure and use multivariable regression methods to assess the magnitude and significance of associations between this PCEs score and (1) adult depression and/or poor mental health (D/PMH) and (2) adults’ reported social and emotional support (ARSES). Separate assessment of associations was conducted for each of 4 ACEs exposure levels.

## Methods

### Population and Data

Data were from the cross-sectional 2015 WI BRFS, a representative, telephone survey of noninstitutionalized Wisconsin adults 18 years and older who speak English or Spanish (n = 6188).^57^ The WI BRFS response rate was 45.0% (weighted American Association of Public Opinion Research median, 47.2%). The cooperation rate was 64.9% (weighted American Association of Public Opinion Research median, 68.0%). The 2015 WI BRFS core and state-added items data sets were linked. Institutional review board (IRB) approval was not required because data are based on a survey conducted by a public agency and do not include personal health information. Respondent oral consent methods and construction of race/ethnicity variables used standard CDC BRFS approved methods.

There were 18.1% to 21.1% missing cases for state-added ARSES, ACEs, and PCEs items. “Don’t know/refused” responses to these questions were 0.2% to 0.9%. A 10% missing value rate for the WI BRFS state-added items is expected and is attributed to the administration of the core WI BRFS survey by another state to Wisconsin residents who have out-of-state cellular phones. In these cases, the WI BRFS state-added items were not available to be administered.^59^ The remainder of missing cases were nearly all owing to respondent dropoffs prior to administering the ARSES, ACEs, and PCEs questions after administration of the core WI BRFS. Differences in D/PMH prevalence rates between respondents and missing cases were not notable. See eTable 1 in the Supplement for additional details.

### Key Measures

#### Positive Childhood Experiences Score

The PCEs score included 7 items asking respondents to report how often or how much as a child they: (1) felt able to talk to their family about feelings; (2) felt their family stood by them during difficult times; (3) enjoyed participating in community traditions; (4) felt a sense of belonging in high school (not including those who did not attend school or were home schooled); (5) felt supported by friends; (6) had at least 2 nonparent adults who took genuine interest in them; and (7) felt safe and protected by an adult in their home. The PCEs score items were adapted from 4 subscales included in the Child and Youth Resilience Measure–28 ^60^: (1) 4 items from the Psychological, Caregiving subscale (see PCEs items 1, 2, 7, and 6 listed previously); (2) 1 from the Education subscale (PCEs item 4); (3) 1 from the Culture subscale (PCEs item 3), and (4) 1 from the Peer Support subscale (PCEs item 5). Items were designed in the Child and Youth Resilience Measure–28 for cultural sensitivity, and their validity was supported by associations with improved resilience.^61^ Psychometric analyses confirmed use of a PCEs cumulative score. See eTable 2 in the Supplement for details.

#### Adverse Childhood Experiences

We used data from the standardized ACEs survey items defined by the CDC.^62,63^ The ACEs measure included 11 ACEs items assessing recollections of childhood experiences of physical or emotional abuse or neglect, sexual abuse, and household dysfunctions such as substance abuse, parental incarceration, and divorce. As recommended by the CDC, items were coded using cumulative score groupings of 0, 1, 2 to 3, or 4 to 8 ACEs. Subjective reports of experiences in childhood are the intended construct for assessment of both PCEs and ACEs and not whether what is reported would be validated using objective assessments.^64^

#### Adult-Reported Social and Emotional Support

Adult-reported social and emotional support is assessed using a standardized single item, “How often do you get the social and emotional support you need?” Response choices were “always,” “usually,” “sometimes,” “rarely,” or “never.” Based on previous research and analysis of this ARSES variable, this study separately evaluated “always” and “usually” responses and created a combined “sometimes/rarely/never” response category.^45,47,48^

#### Depression/Poor Mental Health

The D/PMH category was constructed using (1) the single item on depression asking whether a physician or other health professional “ever told you that you have a depressive disorder, including depression, major depression, dysthymia, or minor depression?”; and (2) a score of 14 or higher on the single item validated as an indicator of current poor mental health^59,60,65,66^ that asked, “Now thinking about your mental health, which includes stress, depression, and problems with emotions, for how many days during the past 30 days was your mental health not good?” Adults reporting either or both of these outcomes were included in the D/PMH variable.

#### Other Covariates

Demographic covariates included age (18-34 years, 35-54 years, 55-64 years, and 65 years or older), race/ethnicity (nonwhite or white/non-Hispanic), and annual income (less than $25 000, $25 000-$49 999, $50 000-$74 999, and $75 000 or more). Sample size and statistical power analysis findings required combining race/ethnicity subgroups into 2 categories for purposes of statistical analysis.

### Analytic Methods

Prevalence rates for all variables were computed, and bivariate associations between individual PCE items and PCEs cumulative score groups and all other variables were evaluated using χ^2^ tests. Iterative and recursive analyses confirmed independent variable construction and focused on confirmation of assumptions on the linearity and comparability of associations with study outcomes when ordinal (count) or cumulative score groupings of PCEs and ACEs were used. Cumulative score groups of 0 to 2, 3 to 5, and 6 to 7 PCEs and 0, 1, 2 to 3, and 4 to 8 ACEs were also selected to ensure adequate statistical power to detect meaningful associations. Such score groups also simplify reporting of results by narrowing the number of comparative groups requiring reporting. Interaction variables crossing PCEs by ACEs and PCEs by ARSES were also analyzed for each study outcome and supported decisions to assess PCEs, ACEs, and ARSES as independent (vs interacting) variables in regression models.

As noted, multivariable logistic regression analyses evaluated 2 association pathways between PCEs items and cumulative score groups and 2 outcome variables: (1) meeting criteria for D/PMH and (2) reports of “always” on ARSES. Regression models were adjusted for age, sex, race/ethnicity, income, and ACEs. Separate models were evaluated for each ACEs exposure level (0, 1, 2-3, and 4-8) to examine stability of associations across ACEs exposure levels. We further assessed the stability of associations between D/PMH and PCEs when ARSES were or were not controlled for in regression models. This was done to further understand more nuanced association pathways between PCEs and ARSES and their individual or interacting association with D/MPH. Additional sensitivity analyses of PCEs associations when ACEs were or were not included in models were also conducted. The survey data were weighted to be representative of the Wisconsin population. We used SPSS Complex Samples, version 24 (IBM Corporation) for data analysis.^67^ A *P* value of .05 or less was used to determine statistical significance.

## Results

### Population Characteristics and Prevalence of Study Outcomes by PCEs

**Figure 1.**
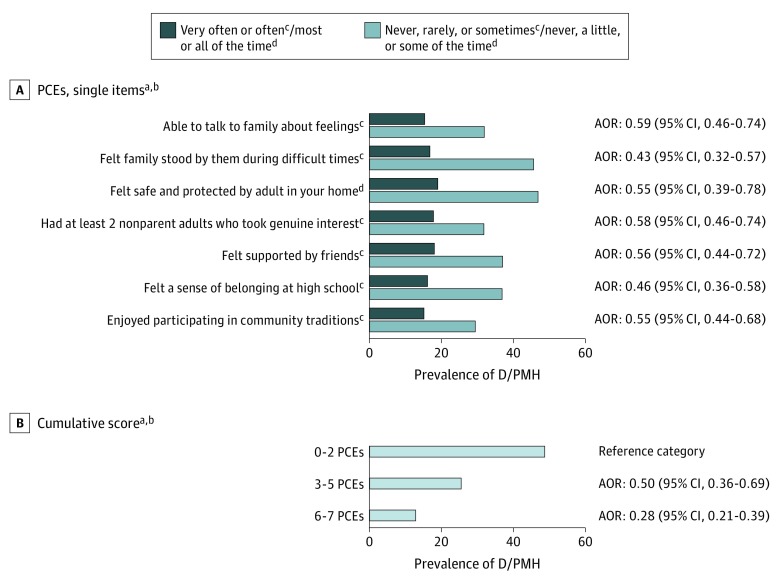
Prevalence of Depression and/or Poor Mental Health Among Adults by Positive Childhood Experiences (PCEs) Single Items and Cumulative Scores See eTable 5 in the Supplement for percentages of depression and/or poor mental health and adult-reported social and emotional support across PCEs items and scores. ^a^Source: authors’ analysis of the 2015 Wisconsin Behavioral Risk Factor Survey. ^b^Adjusted odds ratios (AORs) shown are adjusted for age, sex, race/ethnicity, income, and adverse childhood experiences. ^c^Never, rarely, or sometimes is the reference category. ^d^Never, a little, or some of the time is the reference category.

**Figure 2.**
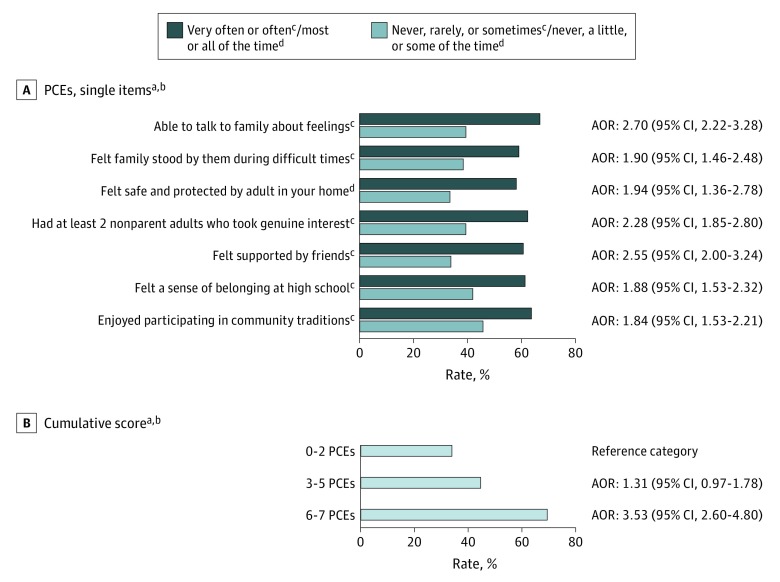
Prevalence of Adult Reporting Always Receiving Needed Social Emotional Support by Positive Childhood Experiences (PCEs) Single Items and Cumulative Scores See eTable 5 in the Supplement for percentages of depression and/or poor mental health and adult-reported social and emotional support across PCEs items and scores. ^a^Source: authors’ analysis of the 2015 Wisconsin Behavioral Risk Factor Survey. ^b^Adjusted odds ratios (AORs) shown are adjusted for age, sex, race/ethnicity, income, and adverse childhood experiences. ^c^Never, rarely, or sometimes is the reference category. ^d^Never, a little, or some of the time is the reference category.

Demographic characteristics for the 2015 WI BRFS mirrored the state population: 50.7% women and 84.9% white. About half (52.3%) reported 6 to 7 PCEs, more than half (56.7%) reported ACEs, 21.2% met D/PMH criteria, and more than half (55.1%) reported “always” to getting the social and emotional support they needed (ARSES). Nonwhite, younger, and lower-income adults reported fewer levels of PCEs (Table 1). Compared with those reporting 6 to 7 PCEs, adults reporting 0 to 2 PCEs had nearly 4 times higher prevalence of D/PMH (48.2% vs 12.6%) and were half as likely to report “always” to getting the social and emotional support they needed (33.0% vs 67.9%) (Table 2). Similar variations in prevalence were observed when each of the 7 PCEs items were separately evaluated for each study outcome (Figures 1 and 2 and eTable 5 in the Supplement). As hypothesized and shown in these Figures, stronger associations emerged for cumulative PCEs scores.

**Table 1.** Study Population Characteristics and Prevalence of PCEs by D/PMH, ACEs, ARSES, and Demographic Characteristics

Population Characteristics (n = Unweighted Sample Size)	Statewide Population Prevalence Estimates		Prevalence of PCEs (n = 4926)^a^						*P* Value (Test of Independence)
			0-2 PCEs		3-5 PCEs		6-7 PCEs		
	Unweighted No.	Weighted %	Unweighted No.	Weighted %	Unweighted No.	Weighted %	Unweighted No.	Weighted %	
All respondents	6188	100	635	13.2	1606	34.5	2685	52.3	NA
D/PMH (n = 6187)									
Yes	1289	21.2	294	29.4	402	40.1	347	30.5	<.001
No	4898	78.8	341	8.7	1204	33.0	2338	58.3	
ACEs exposure levels (n = 4974)^a^^,^^b^									
0 ACEs	2275	43.3	106	4.9	567	27.3	1568	67.8	<.001
1 ACE	1142	23.0	100	8.3	406	38.6	625	53.1	
2-3 ACEs	967	19.9	174	18.5	400	42.1	390	39.5	
4-8 ACEs	590	13.7	255	39.4	232	39.4	100	21.2	
ARSES (n = 5021)^a^									
Always	2707	55.1	195	7.9	687	27.3	1743	64.8	<.001
Usually	1337	25.8	171	12.9	507	41.9	635	45.2	
Sometimes, rarely, or never	977	19.1	263	28.7	393	44.7	284	26.6	
Age (n = 6127), y									
18-34	977	28.8	98	13.0	267	37.9	350	49.2	.03
35-54	1737	33.0	201	15.6	407	31.9	748	52.5	
55-64	1426	17.6	169	12.6	389	36.0	613	51.4	
65 or older	1987	20.5	163	10.4	532	33.1	954	56.5	
Sex (n = 6188)									
Male	2720	49.3	248	11.9	763	36.3	1133	51.7	.09
Female	3468	50.7	387	14.3	843	32.8	1552	52.9	
Race/ethnicity (n = 6129)									
Nonwhite	757	15.1	107	17.0	208	44.7	233	38.3	<.001
White, non-Hispanic	5372	84.9	521	12.6	1385	33.1	2433	54.3	
Income level (n = 5461),^c^ $									
<24 999	1331	22.5	219	22.0	387	38.3	437	39.6	<.001
25 000-49 999	1511	27.8	168	14.9	431	36.9	631	48.3	
50 000-74 999	1010	18.9	83	9.7	288	39.1	465	51.3	
75 000 or more	1609	30.7	105	8.2	334	25.9	888	66.0	

Abbreviations: ACEs, adverse childhood experiences; ARSES, adult-reported social and emotional support; D/PMH, depression and/or poor mental health; NA, not applicable; PCEs, positive childhood experiences; WI BRFS, Wisconsin Behavioral Risk Factor Survey.

^a^ A 10% missing value rate is expected and attributed to core WI BRFS survey administration to out-of-state cellular phone holders who never received the WI BRFS state added items.^59^ The remainder were nearly all owing to respondent dropoffs prior to administering the ARSES, ACEs, and PCEs questions, which were administered after the end of the core WI BRFS. No notable differences in prevalence of D/PMH were found between respondents and cases missing ARSES, ACEs, or PCEs data. See eTable 1 in the Supplement.

^b^ The ACEs cumulative scores were created placing adults into categories of 0, 1, 2 to 3, or 4 to 8 ACEs based on their responses to the 11 ACEs items. Three sexual abuse items were combined into a single item, and alcohol and substance abuse items were presented as a single ACEs item.

^c^ Income missing values rate was 11.7%.

**Table 2.** Prevalence and Adjusted Odds Ratios of Adult D/PMH and Reports of “Always” on the ARSES Item by PCEs and Other Regression Model Variables

Population Characteristics (Raw Sample Size)	Prevalence of D/PMH		*P* Value	Adjusted Odds Ratio (95% CI) for Meeting D/PMH Criteria	Prevalence of “Always” on ARSES Item		*P* Value	Adjusted Odds Ratio (95% CI) for Reports of “Always” on ARSES Item^a^
	Unweighted No.	Weighted %			Unweighted No.	Weighted %		
**All Respondents**	**1289 **	21.2	NA	NA	**2707 **	55.1	NA	NA
Positive childhood experiences (PCEs) (n = 4926)^a,^^b^^,^^c^								
0-2 PCEs reported	294	48.2	<.001	1 [Reference]	195	33.0	<.001	1 [Reference]
3-5 PCEs reported	402	25.1		0.50 (0.36-0.69)	687	43.6		1.31 (0.97-1.78)
6-7 PCEs reported	347	12.6		0.28 (0.21-0.39)	1743	67.9		3.53 (2.60-4.80)
Adverse childhood experiences (ACEs) (n = 4974)^a^								
No ACEs reported	252	11.9	<.001	1 [Reference]	1394	62.4	<.001	1.22 (0.88-1.69)
1 ACE reported	215	20.2		1.62 (1.18-2.21)	596	53.9		0.93 (0.67-1.30)
2-3 ACEs reported	294	29.2		2.40 (1.77-3.24)	439	47.6		0.90 (0.64-1.27)
4-8 ACEs reported	285	42.4		3.10 (2.20-4.37)	226	44.2		1 [Reference]
Age (n = 6127), y								
18-34	215	21.0	.01	1.09 (0.78-1.53)	408	56.8	.44	1.09 (0.84-1.42)
35-54	406	22.6		1.51 (1.10-2.06)	766	54.9		0.97 (0.76-1.23)
55-64	331	24.2		1.64 (1.20-2.24)	600	52.1		0.88 (0.69-1.13)
65 or older	332	16.9		1 [Reference]	911	55.8		1 [Reference]
Sex (n = 6188)								
Male	444	16.9	<.001	0.59 (0.47-0.74)	1189	55.3	.80	0.97 (0.81-1.17)
Female	845	25.5		1 [Reference]	1518	54.8		1 [Reference]
Race/ethnicity (n = 6129)								
Nonwhite	203	23.8	<.25	0.98 (0.67-1.42)	294	53.5	.64	1.19 (0.84-1.70)
White, non-Hispanic	1078	20.9		1 [Reference]	2391	55.2		1 [Reference]
Income level (n = 5461),^d^ $								
<24 999	454	33.3	<.001	2.91 (2.11-4.02)	465	47.8	<.001	0.67 (0.51-0.88)
25 000-49 999	340	22.6		1.76 (1.29-2.41)	667	53.4		0.81 (0.64-1.03)
50 000-74 999	172	18.4		1.43 (1.02-2.01)	458	54.3		0.81 (0.62-1.05)
75 000 or more	205	13.1		1 [Reference]	857	62.3		1 [Reference]

Abbreviations: ACEs, adverse childhood experiences; ARSES, adult-reported social and emotional support; D/PMH, depression and/or poor mental health; NA, not applicable; PCEs, positive childhood experiences; WI BRFS, Wisconsin Behavioral Risk Factor Survey.

^a^ A 10% missing value rate is expected and attributed to core WI BRFS 5 survey administration to out-of-state cellular phone holders who never received the WI BRFS state added items.^59^ The remainder were nearly all owing to respondent dropoffs prior to administering the ARSES, ACEs, and PCEs questions, which were administered after the end of the core WI BRFS. No notable differences in prevalence of D/PMH were found between respondents and cases missing ARSES, ACEs, or PCEs data. See eTable 1 in the Supplement.

^b^ Without adjustment for ACEs, PCEs associations with D/PMH were 0.19 (95% CI, 0.14-0.25) and 0.40 (95% CI, 0.30-0.54) for adults reporting 6 to 7 and 3 to 5 PCEs vs 0 to 2 PCEs, respectively.

^c^ Without adjustment for ACEs, PCEs associations with “always” on the ARSES variable were 3.83 (95% CI, 2.89-5.06) and 1.35 (95% CI, 1.01-1.81) for adults reporting 6 to 7 and 3 to 5 PCEs vs 0 to 2 PCEs, respectively.

^d^ Income missing values rate is 11.7%. Income was not imputed for the WI BRFS by the Wisconsin Department of Health Services so federal poverty level could not be calculated.

The lowest adult D/PMH prevalences were observed for respondents reporting both 6 to 7 PCEs and either no ACEs (10.5%) or “always” on the ARSES variable (8.5%). Highest D/PMH prevalences were for those reporting 0 to 2 PCEs and either 4 to 8 ACEs (59.7%) or “sometimes/ rarely/never” on the ARSES variable (61.7%). Yet, even among those reporting always getting needed social and emotional support, a subset reported 0 to 2 PCEs, and this group had 4 times greater prevalence of D/PMH compared with those reporting 6 to 7 PCEs (33.8% vs 8.5%). Likewise, 21.2% of those with 4 to 8 ACEs and 26.6% of those reporting “sometime/rarely/never” to the ARSES item nonetheless also reported 6 to 7 PCEs. (Table 1, Table 3, and eTable 3 in the Supplement).

**Table 3.** Prevalence of D/PMH and Reports of “Always” on the ARSES Item by PCEs Scores for Each of 4 Adverse Childhood Experiences ACEs Exposure Levels (0, 1, 2-3, or 4-8)

Categories by ACEs and PCEs	Meets D/PMH Criteria^a^			Reports of “Always” to Getting Needed Social and Emotional Support (ARSES)		
	Unweighted No.	Weighted %	Adjusted Odds Ratio^b^ (95% CI)	Unweighted No.	Weighted %	Adjusted Odds Ratio^b^ (95% CI)
No ACEs reported						
0-2 PCEs	17	12.1	1 [Reference]	35	34.6	1 [Reference]
3-5 PCEs	86	15.8	1.15 (0.51-2.62)	266	47.3	1.58 (0.84-2.95)
6-7 PCEs	148	10.5	0.88 (0.42-1.87)	1072	70.5	4.18 (2.31-7.55)
1 ACE reported						
0-2 PCEs	35	45.7	1 [Reference]	38	30.9	1 [Reference]
3-5 PCEs	85	24.2	0.38 (0.17-0.83)	161	39.5	1.33 (0.68-2.62)
6-7 PCEs	94	13.4	0.21 (0.10-0.46)	390	67.6	4.93 (2.54-9.58)
2-3 ACEs reported						
0-2 PCEs	87	53.3	1 [Reference]	47	30.3	1 [Reference]
3-5 PCEs	131	31.4	0.47 (0.26-0.84)	167	43.9	1.65 (0.90-3.02)
6-7 PCEs	76	16.0	0.18 (0.10-0.34)	223	59.2	2.80 (1.53-5.13)
4-8 ACEs reported						
0-2 PCEs	155	59.7	1 [Reference]	75	35.1	1 [Reference]
3-5 PCEs	100	36.9	0.49 (0.28-0.84)	93	41.7	1.19 (0.69-2.03)
6-7 PCEs	29	20.7	0.23 (0.11-0.46)	56	65.6	3.37 (1.66-6.84)

Abbreviations: ACEs, adverse childhood experiences; ARSES, adult-reported social and emotional support; D/PMH, depression and/or poor mental health; PCEs, positive childhood experiences.

^a^ Prevalence of D/PMH varied across levels of ACEs within each PCEs cumulative score category (0-2, 3-5, and 6-7) at *P* < .01.

^b^ Adjusted odds ratios adjusted for age, sex, race/ethnicity, and income.

### Association Pathway 1: PCEs and D/PMH

After controlling for ACEs, the adjusted odds of D/PMH were 72% lower (odds ratio [OR], 0.28; 95% CI, 0.21-0.39) for adults with the highest vs lowest PCEs scores (12.6% vs 48.2%). Odds were 50% lower (OR, 0.50; 95% CI, 0.36-0.69) for those reporting intermediate PCEs scores of 3 to 5 (25.1% vs 48.2%) (Table 2). Associations were similar in magnitude for adults reporting 1, 2 to 3, or 4 to 8 ACEs (Table 3).

### Association Pathway 2: PCEs and ARSES

The adjusted odds of “always” reports on the ARSES item were 3.53 times (95% CI, 2.60-4.80) greater for adults with the highest vs lowest PCEs scores. Adjusted odds of reports of “always” on the ARSES variable were not significant for adults with intermediate PCEs of 3 to 5 (adjusted OR, 1.31; 95% CI, 0.97-1.78) (Table 2). Findings were similar across all ACEs exposure level subgroups (Table 3). Because PCEs and ARSES were strongly associated as hypothesized, we further examined whether each variable demonstrated an independent association with D/PMH and whether associations of PCEs with D/PMH remained stable when ARSES was included in regression models. Results showed that PCEs associations with D/PMH remained significant and changed only modestly when ARSES was included. Associations between ARSES and D/PMH also remained stable when PCEs were or were not included. See eTable 4 in the Supplement for details.

## Discussion

This study examined the prevalence of adult reports of both PCEs and ACEs in a statewide sample and found that PCEs both co-occur with and operate independently from ACEs in their associations with the adult health outcomes evaluated here. Findings also confirm the hypotheses that PCEs may exert their association with D/PMH through their association with ARSES. However, PCEs maintained an association with D/PMH independent from ARSES. Findings are both consistent with prior research showing that relational experiences in childhood are associated with adult social and relational skills and health^3,15,56,68^ and also point to enduring effects of PCEs on D/PMH separate from their influence on adult ARSES.

While PCEs associations with D/PMH were substantial and similar for adults reporting ACEs, associations were not statistically significant for those reporting no ACEs. Insignificant findings may be owing to low sample sizes for respondents with no ACEs and fewer PCEs. Results still raise questions for further exploration. We hypothesize that PCEs may have a greater influence in promoting positive health, such as getting needed social and emotional support or flourishing as an adult. In turn, these positive health attributes may reduce the burden of illness even if the illness is not eliminated. This is consistent with prior research demonstrating a dual continuum of health whereby flourishing is found to be present for many adults despite concurrent mental health conditions.^69^

### Limitations

First, this study is cross-sectional and cannot confirm causal effects. Second, the 2015 Wisconsin adult population is less diverse than the United States as a whole. Third, PCEs focused on the domain of positive emotional experiences in interpersonal relationships. Other types of positive experiences, (eg, safe and supportive environments, nature or spiritual experiences, participation in activities, or accomplishment) require further study, highlighting the need to develop and test additional measures of PCEs. Fourth, we were not able to directly examine bias in reporting of PCEs among adults with depression, although studies show an absence of such biases for reports of ACEs.^64,70^ Finally, the WI BRFS did not assess overall well-being or flourishing.^69^ As such, we were not able to assess whether PCEs affect positive adult health outcomes as hypothesized. Sample size limitations may have resulted in false-negative findings in some cases.

## Conclusions

Overall, study results demonstrate that PCEs show a dose-response association with adult mental and relational health, analogous to the cumulative effects of multiple ACEs. Findings suggest that PCEs may have lifelong consequences for mental and relational health despite co-occurring adversities such as ACEs. In this way, they support application of the World Health Organization’s definition of health emphasizing that health is more than the absence of disease or adversity.^71^ The World Health Organization’s positive construct of health is aligned with the proactive promotion of positive experiences in childhood because they are foundational to optimal childhood development and adult flourishing. Including PCEs as well as positive health outcomes measures in routinely collected public health surveillance systems, such as the National Survey of Children’s Health and state Behavioral Risk Factor Surveillance Surveys, may advance knowledge and allow the nation to track progress in promoting flourishing despite adversity or illness among children and adults in the United States.

Even as society continues to address remediable causes of childhood adversities such as ACEs, attention should be given to the creation of those positive experiences that both reflect and generate resilience within children, families, and communities. Success will depend on full engagement of families and communities and changes in the health care, education, and social services systems serving children and families. A joint inventory of ACEs and PCEs, such as the positive experiences assessed here, may improve efforts to assess needs, target interventions, and engage individuals in addressing the adversities they face by leveraging existing assets and strengths.^72^ Initiatives to conduct broad ACEs screening, such as those ensuing in California’s Medicaid program, may benefit from integrated assessments including PCEs.^73^

Recommendations and practice guidelines included in the *National Bright Futures Guidelines for Health Supervision of Infants, Children, and Adolescents*^74^ and the CDC’s *Essentials for Childhood* initiative^9^ encourage policies and initiatives to help child-serving professionals and programs to adopt effective approaches to promote the type of PCEs evaluated in this study. The *Health Outcomes of Positive Experiences* framework^48^ and the Prioritizing Possibilities national agenda for promoting child health and addressing ACEs^75^ each seek to advance existing and emerging evidence-based approaches^44,45,47,48,50,54,76,77^ that promote a positive construct of health in clinical, public health, and human services settings. This study adds to the growing evidence that childhood experiences have profound and lifelong effects. Results hold promise for national, state, and community efforts to achieve positive child and adult health and well-being by promoting the largely untapped potential to promote positive experiences and flourishing despite adversity.^53,78^

## Notes

Supplement. **eTable 1.** Prevalence of Depression/Poor Mental Health (D/PMH) and Demographic Characteristics Among Adults With or Without Missing Values for Positive Childhood Experiences (PCEs), Adult Reported Social and Emotional Support (ARSES) and Adverse Childhood Experiences (ACEs) WI BRFS State Added Items **eTable 2.** Summary of Findings From Psychometric Analyses^a^ Conducted on Positive Childhood Experiences (PCEs) Seven Item Measure **eTable 3.** Prevalence of Adult Depression and/or Poor Mental Health (D/PMH) by Adult Reported Social and Emotional Support (ARSES) and Positive Childhood Experiences (PCEs) **eTable 4.** Adjusted Odds Ratios of Adult Depression and/or Poor Mental Health (D/PMH) by Adult Reported Social and Emotional Support (ARSES) and Positive Childhood Experiences (PCEs) Under Alternative Model Configurations **eTable 5.** Prevalence of Depression and/or Poor Mental Health (D/PMH) and Adult Reporting "Always" Receiving Needed Social Emotional Support (ARSES), By Positive Childhood Experiences (PCEs) Cumulative Scores and Single Items
