# Supplementary material for: Positive Childhood Experiences and Adult Mental and Relational Health in a Statewide Sample: Associations Across Adverse Childhood Experiences Levels
Source: JAMA Pediatr. 2019 Sep 9;173(11):e193007. doi: 10.1001/jamapediatrics.2019.3007 (PMC6735495; doi:10.1001/jamapediatrics.2019.3007)
Supplement: Supplement. — eTable 1. Prevalence of Depression/Poor Mental Health (D/PMH) and Demographic Characteristics Among Adults With or Without Missing Values for Positive Childhood Experiences (PCEs), Adult Reported Social and Emotional Support (ARSES) and Adverse Childhood Experiences (ACEs) WI BRFS State Added Items eTable 2. Summary of Findings From Psychometric Analysesa Conducted on Positive Childhood Experiences (PCEs) Seven Item Measure eTable 3. Prevalence of Adult Depression and/or Poor Mental Health (D/PMH) by Adult Reported Social and Emotional Support (ARSES) and Positive Childhood Experiences (PCEs) eTable 4. Adjusted Odds Ratios of Adult Depression and/or Poor Mental Health (D/PMH) by Adult Reported Social and Emotional Support (ARSES) and Positive Childhood Experiences (PCEs) Under Alternative Model Configurations eTable 5. Prevalence of Depression and/or Poor Mental Health (D/PMH) and Adult Reporting "Always" Receiving Needed Social Emotional Support (ARSES), By Positive Childhood Experiences (PCEs) Cumulative Scores and Single Items [file jamapediatr-173-e193007-s001.pdf]

Supplementary Online Content

Bethell C, Jones J, Gombojav N, Linkenbach J, Sege R. Positive childhood experiences and adult mental and relational health in a statewide sample: associations across adverse childhood experiences levels. *JAMA Pediatr.* Published online September 9, 2019. 10.1001/jamapediatrics.2019.3007

eTable 1. Prevalence of Depression/Poor Mental Health (D/PMH) and Demographic Characteristics Among Adults With or Without Missing Values for Positive Childhood Experiences (PCEs), Adult Reported Social and Emotional Support (ARSES) and Adverse Childhood Experiences (ACEs) WI BRFS State Added Items

eTable 2. Summary of Findings From Psychometric Analyses[a] Conducted on Positive Childhood Experiences (PCEs) Seven Item Measure

eTable 3. Prevalence of Adult Depression and/or Poor Mental Health (D/PMH) by Adult Reported Social and Emotional Support (ARSES) and Positive Childhood Experiences (PCEs)

eTable 4. Adjusted Odds Ratios of Adult Depression and/or Poor Mental Health (D/PMH) by Adult Reported Social and Emotional Support (ARSES) and Positive Childhood Experiences (PCEs) Under Alternative Model Configurations

eTable 5. Prevalence of Depression and/or Poor Mental Health (D/PMH) and Adult Reporting "Always" Receiving Needed Social Emotional Support (ARSES), By Positive Childhood Experiences (PCEs) Cumulative Scores and Single Items

© 2019 Bethell C et al. *JAMA Pediatrics.*

eTable 1: Prevalence of depression/poor mental health (D/PMH) and demographic characteristics among adults with or without missing values for positive childhood experiences (PCEs), adult reported social and emotional support (ARSES) and adverse childhood experiences (ACEs) WI BRFS state added items.

	**Positive childhood experiences (PCEs)**		**Adult reported social and emotional support (ARSES)**		**Adverse childhood experiences (ACEs)**	
	Respondents	Non-respondents[a]	Respondents	Non-respondents[a]	Respondents	Non-respondents[a]
All adults	Raw n=4926	Raw n=1256	Raw n=5021	Raw n=1117	Raw n=4974	Raw n=1200
Depression/poor mental health (D/PMH) (n=6187)						
Yes	21.6%	20.2%	21.5%	20.5%	21.5%	20.6%
No	78.4%	79.8%	78.5%	79.5%	78.5%	79.4%
Age (n=6127)						
18-34 years	26.4%	38.3%	26.4%	37.5%	26.3%	37.5%
35-54 years	32.9%	33.2%	32.9%	33.4%	32.9%	33.4%
55-64 years	18.7%	13.9%	18.6%	14.2%	18.7%	14.2%
65 or older	22.0%	14.6%	22.1%	14.8%	22.2%	14.8%
Sex (n=6188)						
Male	49.1%	49.9%	49.2%	49.1%	49.1%	49.8%
Female	50.9%	50.1%	50.8%	50.9%	50.9%	50.2%
Race/ethnicity (n=6129)						
Non-White	12.4%	23.8%	12.3%	25.1%	12.3%	24.5%
White, Non-Hispanic	87.6%	76.2%	87.7%	74.9%	87.7%	75.5%
Income level (n=5461)[b]						
<$24,999	20.6%	29.3%	20.6%	29.9%	20.6%	29.3%
$25,000-$49,999	27.9%	27.4%	27.9%	27.8%	27.9%	27.4%
$50,000-$74,999	19.8%	15.7%	19.6%	16.0%	19.8%	15.9%
$75,000 or more	31.7%	27.5%	31.9%	26.3%	31.7%	27.5%

Source: Author's analysis of the 2015 Wisconsin Behavioral Risk Factor Survey (BRFS).

[a] A 10% missing value rate is expected and attributed to core WI BRFS survey administration to out-of-state cell phones who never received the WI BRFS state added items.[59] The remainder were nearly all due to respondent drop-offs prior to administering the ARSES, ACEs and PCEs questions, which were administered after the end of the core WI BRFS. No notable differences in prevalence of D/PMH were found between respondents and cases missing ARSES, ACEs or PCEs data.[b] See eTable 1. Analysis of missing values did not reveal difference in prevalence of D/PMH between respondents and non-respondents. Younger, non-white adults with lower income more likely to have missing values. (see eTable 1). However, study findings less likely to be affected by the missing cases due to adjustments of demographics characteristics in logistic regression analyses. [b]Income missing values rate is 11.7%. Income was not imputed for the Wisconsin BRFS by the Wisconsin Department of Health Services so Federal Poverty Level could not be calculated.

© 2019 Bethell C et al. *JAMA Pediatrics.*

eTable 2: Summary of findings from psychometric analyses[a] conducted on positive childhood experiences (PCEs) seven item measure

Internal Consistency (Cronbach's alpha)	Principle components factor analysis results	Factor loading across seven items
0.77	A single Eigen value greater than 1.0 (2.95) that explained 42.2% of variance	0.57 ("felt safe/home") to<br>0.72 ("family stood by/difficult times")

Source: Author's analysis of the 2015 Wisconsin Behavioral Risk Factor Survey (BRFS).
[a]Assessment of psychometric properties on the use of the 7 PCEs items as a cumulative score measure included correlational analysis, internal consistency reliability and principal components factor analysis. See table above for summary of findings. Analyses also included assessment of the convergent and divergent validity of using a cumulative score version of the PCEs items versus each item separately. Here, we compared associations with study outcomes using the PCEs cumulative scoring approach against associations using each PCEs item separately. See Figures 1 and 2 in the main paper for findings.

eTable 3: Prevalence of adult depression and/or poor mental health (D/PMH) by adult reported social and emotional support (ARSES) and positive childhood experiences (PCEs)

	Statewide population prevalence estimates		Meets depression and/or poor mental health (D/PMH) criteria	
	Raw n	%	%	Adjusted odds ratio[a] (95% CIs)
Reported "always" get needed support				
0-2 PCEs	195	7.9%	33.8%	0.27 (0.15-0.49)
3-5 PCEs	687	27.3%	19.7%	0.19 (0.11-0.31)
6-7 PCEs	1743	64.8%	8.5%	0.10 (0.06-0.16)
Reported "usually" get needed support				
0-2 PCEs	171	12.9%	46.0%	0.48 (0.26-0.89)
3-5 PCEs	507	41.9%	28.2%	0.32 (0.19-0.55)
6-7 PCEs	635	45.2%	19.8%	0.26 (0.16-0.42)
2 Reported "sometimes, rarely or never" get needed support				
0-2 PCEs	263	28.7%	61.7%	Ref (1.00)
3-5 PCEs	393	44.7%	31.5%	0.36 (0.22-0.58)
6-7 PCEs	284	26.6%	25.1%	0.28 (0.15-0.50)

Source: Author's analysis of the 2015 Wisconsin Behavioral Risk Factor Survey (BRFS). [a]Adjusted odds ratios (AORs) AORs adjusted for age, sex, race/ethnicity, income and adverse childhood experiences (ACEs).

eTable 4: Adjusted odds ratios of adult depression and/or poor mental health (D/PMH) by adult reported social and emotional support (ARSES) and positive childhood experiences (PCEs) under alternative model configurations

	Adjusted odds ratios[a] without ARSES adjustment in the model	Adjusted odds ratios[a] without PCEs in the model	Adjusted odds ratios[a] with PCEs and ARSES in the model
Adult reported social and emotional support (ARSES) (n=5021)			
Always	N/A	0.32 (0.24-0.42)	0.38 (0.29-0.51)
Usually	N/A	0.70 (0.51-0.95)	0.79 (0.58-1.08)
Sometimes, rarely or never	N/A	Ref (1.00)	Ref (1.00)
Positive childhood experiences (PCEs) (n=4926)			
0-2 PCEs reported	Ref (1.00)	N/A	Ref (1.00)
3-5 PCEs reported	0.50 (0.36-0.69)	N/A	0.53 (0.39-0.74)
6-7 PCEs reported	0.28 (0.21-0.39)	N/A	0.36 (0.26-0.50)
Age (n=6127)			
18-34 years	1.09 (0.78-1.53)	1.15 (0.81-1.62)	1.15 (0.82-1.62)
35-54 years	1.51 (1.10-2.06)	1.61 (1.17-2.20)	1.54 (1.12-2.12)
55-64 years	1.64 (1.20-2.24)	1.66 (1.21-2.29)	1.64 (1.18-2.27)
65 or older	Ref (1.00)	Ref (1.00)	Ref (1.00)
Sex (n=6188)			
Male	0.59 (0.47-0.74)	0.57 (0.45-0.72)	0.56 (0.44-0.71)
Female	Ref (1.00)	Ref (1.00)	Ref (1.00)
Race/ethnicity (n=6129)			
Non-White	0.98 (0.67-1.42)	1.04 (0.70-1.55)	1.03 (0.69-1.52)
White, Non-Hispanic	Ref (1.00)	Ref (1.00)	Ref (1.00)
Income Level (n=5461)			
<$24,999	2.91 (2.11-4.02)	3.06 (2.21-4.24)	2.71 (1.95-3.77)
$25,000-$49,999	1.76 (1.29-2.41)	1.84 (1.34-2.52)	1.69 (1.23-2.32)
$50,000-$74,999	1.43 (1.02-2.01)	1.38 (0.98-1.95)	1.35 (0.96-1.91)
$75,000 or more	Ref (1.00)	Ref (1.00)	Ref (1.00)
Adverse childhood experiences (ACEs) (n=4974)			
0 ACEs	Ref (1.00)	Ref (1.00)	Ref (1.00)
1 ACE	1.62 (1.18-2.21)	1.65 (1.21-2.25)	1.51 (1.10-2.06)
2-3 ACEs	2.40 (1.77-3.24)	2.74 (2.02-3.73)	2.27 (1.69-3.09)
4-8 (4+) ACEs	3.10 (2.20-4.37)	4.30 (3.10-5.98)	2.99 (2.11-4.22)

Source: Author's analysis of the 2015 Wisconsin Behavioral Risk Factor Survey (BRFS).

© 2019 Bethell C et al. *JAMA Pediatrics.*

eTable 5: Prevalence of depression and/or poor mental health (D/PMH) and adult reporting "always" receiving needed social emotional support (ARSES), by positive childhood experiences (PCEs) cumulative scores and single items. Adjusted Odds Ratios shown[a]

Positive Childhood Experiences (PCEs) cumulative scores and items	Depression and/or poor mental health (D/PMH)			Adult reporting "always" receiving needed social emotional support (ARSES)		
	Raw n	%	Adjusted odds ratio[a] (95% CIs)	Raw n	%	Adjusted odds ratio[a] (95% CIs)
**Positive Childhood Experiences (PCEs) cumulative scores**						
0-2 PCEs	294	48.2%	Reference	195	33.0%	Reference
3-5 PCEs	402	25.1%	0.50 (0.36-0.69)	687	43.6%	1.31 (0.97-1.78)
6-7 PCEs	347	12.6%	0.28 (0.21-0.39)	1743	67.9%	3.53 (2.60-4.80)
**Positive Childhood Experiences (PCEs) items**						
1. Able to talk to family about feelings						
Never, rarely, or sometimes	614	31.7%	Reference	763	38.6%	Reference
Very often or often	422	15.2%	0.59 (0.46-0.74)	1833	65.5%	2.70 (2.22-3.28)
2. Felt family stood by them during difficult times						
Never, rarely, or sometimes	363	45.2%	Reference	282	37.8%	Reference
Very often or often	667	16.7%	0.43 (0.32-0.57)	2307	58.3%	1.90 (1.46-2.48)
3. Felt safe and protected by adult in your home						
Never, a little or some the of time	189	46.7%	Reference	132	33.2%	Reference
Most or all of the time	848	19.0%	0.55 (0.39-0.78)	2486	57.2%	1.94 (1.36-2.78)
4. Had at least 2 non-parent adults who took genuine interest						
Never, rarely, or sometimes	418	31.6%	Reference	507	38.6%	Reference
Very often or often	614	17.6%	0.58 (0.46-0.74)	2080	61.4%	2.28 (1.85-2.80)
5. Felt supported by friends						
Never, rarely, or sometimes	357	36.8%	Reference	304	33.6%	Reference
Very often or often	678	17.9%	0.56 (0.44-0.72)	2301	59.8%	2.55 (2.00-3.24)
6. Felt a sense of belonging at high school						
Never, rarely, or sometimes	443	36.5%	Reference	467	41.3%	Reference
Very often or often	581	16.0%	0.46 (0.36-0.58)	2106	60.3%	1.88 (1.53-2.32)
7. Enjoyed participating in community traditions						
Never, rarely, or sometimes	579	29.2%	Reference	887	45.2%	Reference
Very often or often	446	15.1%	0.55 (0.44-0.68)	1692	62.8%	1.84 (1.53-2.21)

Source: Author's analysis of the 2015 Wisconsin Behavioral Risk Factor Survey (BRFS).
[a]Adjusted odds ratios (AORs) shown are adjusted for age, sex, race/ethnicity, income, and adverse childhood experiences (ACEs).

© 2019 Bethell C et al. *JAMA Pediatrics.*
